# Biventricular longitudinal strain as a predictor of functional improvement after D-shant device implantation in patients with heart failure

**DOI:** 10.3389/fcvm.2023.1121689

**Published:** 2023-04-17

**Authors:** Yi Zhou, He Li, Lingyun Fang, Wenqian Wu, Zhenxing Sun, Ziming Zhang, Manwei Liu, Jie Liu, Lin He, Yihan Chen, Yuji Xie, Yuman Li, Mingxing Xie

**Affiliations:** ^1^ Department of Ultrasound, Union Hospital, Tongji Medical College, Huazhong University of Science and Technology, Wuhan, China; ^2^ Clinical Research Center for Medical Imaging in Hubei Province, Wuhan, China; ^3^ Hubei Province Key Laboratory of Molecular Imaging, Wuhan, China

**Keywords:** HFpEF, HFrEF, D-Shant device, speckle-tracking echocardiography, NYHA functional class

## Abstract

**Background:**

The creation of an atrial shunt is a novel approach for the management of heart failure (HF), and there is a need for advanced methods for detection of cardiac function response to an interatrial shunt device. Ventricular longitudinal strain is a more sensitive marker of cardiac function than conventional echocardiographic parameters, but data on the value of longitudinal strain as a predictor of improvement in cardiac function after implantation of an interatrial shunt device are scarce. We aimed to investigate the exploratory efficacy of the D-Shant device for interatrial shunting in treating heart failure with reduced ejection fraction (HFrEF) and heart failure with preserved ejection fraction (HFpEF), and to explore the predictive value of biventricular longitudinal strain for functional improvement in such patients.

**Methods:**

A total of 34 patients were enrolled (25 with HFrEF and 9 with HFpEF). All patients underwent conventional echocardiography and two-dimensional speckle tracking echocardiogram (2D-STE) at baseline and 6 months after implantation of a D-Shant device (WeiKe Medical Inc., WuHan, CN). Left ventricular global longitudinal strain (LVGLS) and right ventricular free wall longitudinal strain (RVFWLS) were evaluated by 2D-STE.

**Results:**

The D-Shant device was successfully implanted in all cases without periprocedural mortality. At 6-month follow-up, an improvement in New York Heart Association (NYHA) functional class was observed in 20 of 28 patients with HF. Compared with baseline, patients with HFrEF showed significant reduced left atrial volume index (LAVI) and increased right atrial (RA) dimensions, as well as improved LVGLS and RVFWLS, at 6-month follow-up. Despite reduction in LAVI and increase in RA dimensions, improvements in biventricular longitudinal strain did not occur in HFpEF patients. Multivariate logistic regression demonstrated that LVGLS [odds ratio (OR): 5.930; 95% CI: 1.463–24.038; *P* = 0.013] and RVFWLS (OR: 4.852; 95% CI: 1.372–17.159; *P* = 0.014) were predictive of improvement in NYHA functional class after D-Shant device implantation.

**Conclusion:**

Improvements in clinical and functional status are observed in patients with HF 6 months after implantation of a D-Shant device. Preoperative biventricular longitudinal strain is predictive of improvement in NYHA functional class and may be helpful to identify patients who will experience better outcomes following implantation of an interatrial shunt device.

## Introduction

Heart failure (HF), whether it occurs with preserved ejection fraction (HFpEF; defined by a left ventricular ejection fraction [LVEF] ≥40%) or with reduced ejection fraction (HFrEF; defined by a LVEF <40%), remains a major public health care burden associated with substantial morbidity and mortality (1). Although HFpEF and HFrEF are heterogeneous with respect to etiology and pathophysiology, elevation of left atrial pressure (LAP) is the common mechanism precipitating worsening symptoms and acute decompensation (2–5). LA decompression, with the goal of limiting the increase in pulmonary venous pressure, may contribute to improved symptoms and outcomes in these patients (6). Percutaneously implanted permanent interatrial shunt devices have recently been developed for the treatment of patients with HF and have produced encouraging early clinical and hemodynamic results (7–12). Identification of quantitative predictors of the impact of an interatrial shunt device on cardiac functional recovery is crucial and may allow appropriate selection of patients. The clinical efficacy of advanced echocardiographic imaging, such as two-dimensional speckle tracking echocardiography (2D-STE), has been supported by various investigations. A number of studies from the past few years have shown that assessing global longitudinal strain may assist in refining the decision-making process in patients with HF (13–17). However, the clinical value of biventricular longitudinal strain, measured *via* 2D-STE, in HF patients following treatment with an interatrial shunt device has not been described. The objective of this study was to determine the exploratory efficacy of the D-Shant device (WeiKe Medical Inc., WuHan, CN) for treatment of patients with HFrEF and HFpEF, and to assess the predictive value of biventricular longitudinal strain in such patients.

## Methods

### Study population

A total of 34 patients with HFrEF and HFpEF were admitted to the Union Hospital, Tongji Medical College, Huazhong University of Science and Technology in 2020 and 2021. The study was approved by the Ethics Committee of Union Hospital Tongji Medical College, Huazhong University of Science and Technology (20200165). All patients provided written informed consent. The inclusion criteria were: HFpEF (LVEF ≥40%) or HFrEF (LVEF <40%); pulmonary capillary wedge pressure (PCWP) ≥15 mmHg at rest; and New York Heart Association (NYHA) functional class II–IV with chronic heart failure, still experiencing symptoms after at least 4 weeks of standardized drug treatment. The exclusion criteria were: moderate or greater right ventricular (RV) dysfunction; severe liver and kidney damage; recent history of surgery or severe trauma; autoimmune diseases or other serious systemic diseases.

### Clinical data

Demographic characteristics, data from laboratory examinations, and comorbidities of the patients were obtained from electronic medical records. The hemodynamics of PCWP, mean right atrial pressure (RAP), and PCWP-RAP were measured using a fluid-filled balloon-tipped catheter. Exercise capacity was assessed using the 6-min walk test (6MWT). Functional status and quality of life were assessed by evaluation of NYHA functional class and administration of the Kansas City Cardiomyopathy Questionnaire (KCCQ).

### Intervention

**Figure 1:**
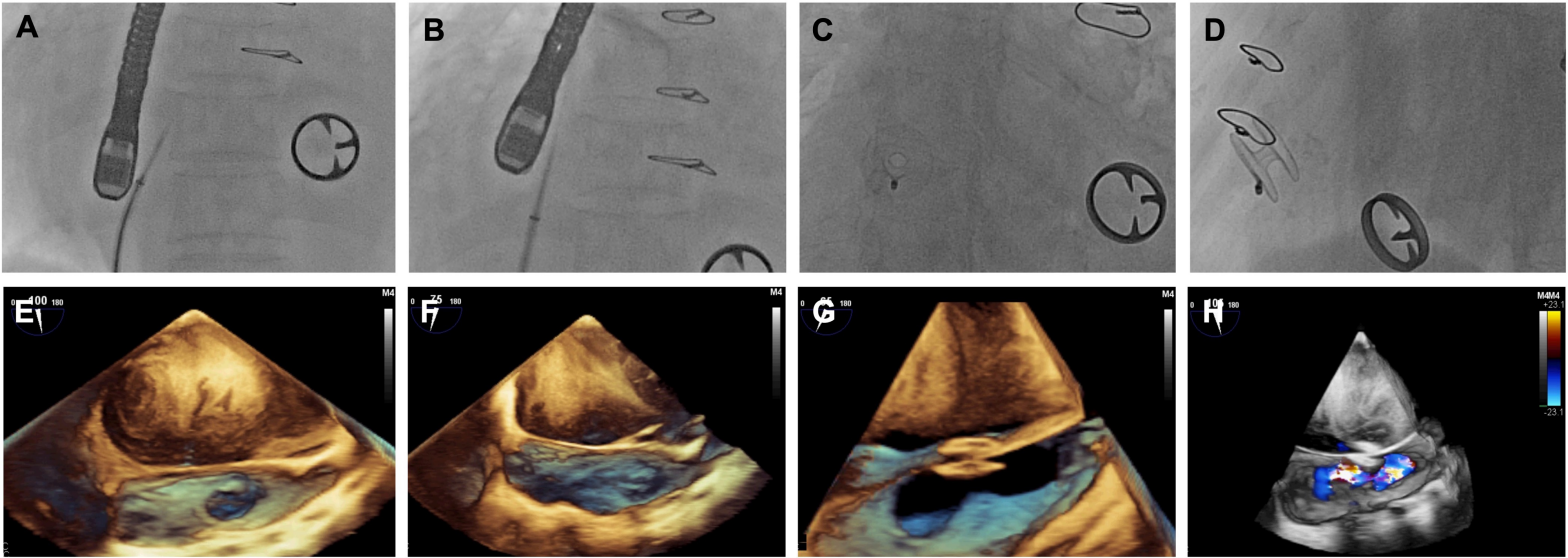
Images of D-shant device implantation. (**A**) x-ray and (**E**) transesophageal echocardiography (TEE) show the atrial septal position; (**B**) x-ray and (**F**) TEE guide atrial septal puncture; (**C**) x-ray and (**G**) TEE show release of the D-Shant device; and finally, (**D**) x-ray and (**H**) TEE color Doppler confirm the position of the D-Shant device and left-to-right shunting.

The procedures were performed under general anesthesia. Standard trans-septal puncture of the interatrial septum was carried out by fluoroscopy and transesophageal echocardiography (Figure 1). The delivery system was advanced *via* the wire into the LA, the left side of the D-Shant device was opened, and the delivery system was retracted to make contact with the septum on the LA side. The right half of the device was then placed into the right atrial side of the septum. The delivery system and guiding wire were then removed.

### Transthoracic echocardiography

Transthoracic echocardiographic examinations were performed in all patients using the EPIQ 7C ultrasound system (Philips Medical Systems, Andover, Massachusetts) at baseline and 6 months after implantation of the D-Shant device. All echocardiographic images were acquired following published American Society of Echocardiography guidelines and analyzed by an experienced investigator who was blinded to the clinical characteristics of the study population. Echocardiographic indices were measured three times, and the mean value was used for the statistical analysis.

### Conventional echocardiography analysis

Conventional echocardiographic parameters were measured following the guidelines of the American Society of Echocardiography (18). LV end-diastolic volume index (LVEDVI), LV end-systolic volume index (LVESVI), and LVEF were measured using the biplane Simpson's method. Mitral inflow peak early diastolic velocity (E), peak late diastolic velocity (A), E/A ratio, septal and lateral mitral annular early diastolic velocities (e′), and the E/e′ ratio were used as indices of diastolic function. Multiple echocardiographic windows were employed to screen for tricuspid regurgitation and spectral Doppler velocity. Left atrial volume index (LAVI) was calculated using the area–length method (19) and indexed to body surface area. RA and RV dimensions were determined from the apical 4-chamber view. Tricuspid annulus plane systolic excursion (TAPSE) was measured as the systolic displacement of the tricuspid lateral annulus, as recorded on M-mode imaging. Right ventricular fractional area change (RVFAC) was calculated from the end-diastolic and end-systolic areas derived from the apical 4-chamber view. Tricuspid lateral annular systolic velocity (S′) was assessed using tissue Doppler imaging from the apical 4-chamber view. Finally, valvular regurgitation severity was graded qualitatively according to current recommendations: none/trace (grade 0), mild (grade I), moderate (grade II), or severe (grade III) (20).

### 2D-STE analysis

2D-STE analysis was conducted according to the recommendations of the American Society of Echocardiography and the European Association of Cardiovascular Imaging (21). Left ventricular global longitudinal strain (LVGLS) and right ventricular free wall longitudinal strain (RVFWLS) were measured using the 2D Auto Strain software package (Qlab13, Philips Healthcare, Andover, Massachusetts). The software tracked the endocardial borders of the left and right ventricles throughout the cardiac cycle, with manual modifications as needed. Images with low tracking quality were excluded. LVGLS was acquired by averaging three apical views (the apical 2-, 3-, and 4-chamber views). RVFWLS was calculated as the mean of the strain values in the three segments of the RV free wall. The average frame rate of the clips used for 2D-STE analysis was 50–70 frames/s.

### Intra- and inter-observer reproducibility

Intra-observer and inter-observer reproducibility for the LVGLS and RVFWLS measurements obtained from 28 patients were assessed *via* calculation of intra-class correlation coefficients (ICCs) and Bland–Altman analysis. Intra-observer reproducibility was analyzed by having the same investigator repeat the biventricular longitudinal strain measurements. Inter-observer reproducibility was determined by comparison of measurements to those obtained by a second investigator who was blinded to the results of the first measurements.

### Statistical analysis

Statistical analysis was performed using IBM SPSS Statistics version 26.0 (SPSS, Inc). Continuous data are reported in the form mean ± SD if normally distributed data or median (interquartile range) if non-normally distributed. Categorical data are reported in the form percentage (number) and were compared using the chi-square test or Fisher's exact test. Measurements of continuous variables obtained at baseline and 6 months after implantation were compared using the paired Student's *t*-test or the Wilcoxon signed rank test. An independent samples t-test or Mann–Whitney *U* test was used to compare the group who exhibited improvement in NYHA functional class and to the group who did not. Univariate and multivariate logistic regression analyses were performed to identify the predictors of improvement in NYHA functional class 6 months after device implantation. Univariate predictors with *P *< 0.10 were included in a multivariate logistic regression, which was used to determine the independent predictors. Receiver operating characteristic (ROC) curve analysis was used to identify the best parameters for prediction of improvement in NYHA functional class. The best cutoff value was based on the maximum Youden index. All statistical tests were two-sided, and a *P* value <0.05 was considered to represent statistical significance.

## Results

### Baseline characteristics

The clinical characteristics of patients with HFrEF and HFpEF at baseline are presented in Table 1. In total, 25 patients with HFrEF and 9 patients with HFpEF were enrolled. The mean age across all patients was 59 ± 11 years, and 18 patients (52.9%) were male. Among all 34 participants, 32 patients (94.1%) were categorized into NYHA functional class III or IV, and the average PCWP was 17.7 ± 4.7 mmHg. Hypertension was present in 7 patients, diabetes in 10 patients, coronary artery disease in 38.2% of patients, and a history of atrial fibrillation or flutter in 4 patients. There were no differences between the HFrEF and HFpEF groups in terms of age, sex, heart rate, systemic or diastolic arterial pressure, incidence of comorbidities (hypertension, diabetes, coronary artery disease, atrial fibrillation or flutter), NYHA functional class, level of hemoglobin, estimated glomerular filtration rate (eGFR), N-terminal pro-B type natriuretic peptide (NT-proBNP), or hemodynamics of the PCWP, RAP, or PCWP-RAP.

**Table 1:** Clinical characteristics of patients with HFrEF and HFpEF at baseline.

	Overall	HFrEF group (*n* = 25)	HFpEF group (*n* = 9)	*P-*value
Demographics				
Age, y	59 ± 11	59 ± 12	60 ± 11	0.730
Male	52.9% (18/34)	52.0% (13/25)	55.6% (5/9)	>0.999
BMI, kg/m^2^	23.0 (20.4, 26.0)	22.7 (19.5, 25.6)	23.5 (21.9, 26.4)	0.140
SBP, mmHg	116 ± 22	115 ± 25	120 ± 10	0.489
DBP, mmHg	75 ± 10	73 ± 11	80 ± 6	0.072
HR, bpm	74 ± 14	74 ± 14	72 ± 15	0.565
Hypertension, % (*n*)	20.6% (7/34)	16.0% (4/25)	33.3% (3/9)	0.348
Diabetes, % (*n*)	29.4% (10/34)	32.0% (8/25)	22.2% (2/9)	0.692
Atrial fibrillation or flutter, % (*n*)	11.8% (4/34)	8.0% (2/25)	22.2% (2/9)	0.281
Coronary artery disease, % (*n*)	38.2% (13/34)	44.0% (11/25)	22.2% (2/9)	0.429
NYHA functional class				0.591
II	5.9% (2/34)	8.0% (2/25)	0.0% (0/9)	
III	76.5% (26/34)	76.0% (19/25)	77.8% (7/9)	
IV	17.6% (6/34)	16.0% (4/25)	22.2% (2/9)	
Laboratory data				
Hemoglobin, g/L	119.5 (106.0, 132.0)	116.0 (101.0, 131.5)	126.0 (110.5, 134.0)	0.298
eGFR, mL/min	80.1 (66.4, 99.3)	81.2 (66.3, 98.9)	75.6 (51.2, 102.9)	0.869
NT-proBNP, pg/mL	1675.4 (403.0, 3795.0)	2100.0 (490.5, 6215.0)	618.0 (358.4, 2005.0)	0.166
Hemodynamics				
PCWP, mmHg	17.7 ± 4.7	18.1 ± 5.2	16.7 ± 2.6	0.818
RAP, mmHg	4.0 (2.0, 7.0)	4.0 (2.0, 7.0)	5.0 (3.0, 5.5)	0.316
PCWP-RAP, mmHg	13.0 (11.0, 15.3)	13.0 (13.0, 17.5)	12.0 (11.5, 14.0)	>0.999

Values are presented in the form mean ± SD, % (*n*), or median (interquartile range). BMI, body mass index; SBP, systolic blood pressure; DBP, diastolic blood pressure; HR, heart rate; NYHA, New York Heart Association; eGFR, estimated glomerular filtration rate; NT-proBNP, N-terminal pro-B type natriuretic peptide; PCWP, pulmonary capillary wedge pressure; RAP, right atrial pressure.

### Clinical and echocardiographic characteristics after implantation

Sizing specifications for the D-Shant device in each patient are shown in Supplementary Table S1. At 6 months after implantation, 5 patients with HFrEF and 1 patient with HFpEF were lost to follow up. Among the remaining patients, there were sustained improvements in NYHA functional class, with 5 patients (17.9%) now in NYHA functional class IV, 6 (21.4%) in NYHA functional class III, 16 (57.1%) in NYHA functional class II, and 1 [3.6%] in NYHA functional class I (Supplementary Figure S1A). Similarly, significant improvements in KCCQ score (32.1 ± 6.5 vs. 80.3 ± 6.6; *P *< 0.001) and 6 MWT results (333.0 ± 53.5 vs. 367.1 ± 74.3; *P *< 0.001) were observed across all patients (Supplementary Figures S1B,C). Table 2 presents the conventional echocardiographic and 2D-STE parameters at baseline and 6 months after implantation. Color flow Doppler imaging confirmed the presence of an interatrial left-to-right shunt at a follow-up visit. With the creation of a small left-to-right shunt, increased RA dimensions were observed among the HFrEF and HFpEF groups. At 6 months after device implantation, a significant reduction in LAVI was found only in the HFrEF group, whereas tricuspid S′, LVEDVI, LVESVI, LVEF, E/A, E/e′, TAPSE, and RVFAC did not differ from baseline values in either the HFrEF or the HFpEF group.

**Table 2:** Echocardiographic characteristics of patients with HFrEF and HFpEF at baseline and 6 months after D-shant device implantation.

Parameter	Overall			HFrEF group (*n* = 20)			HFpEF group (*n* = 8)		
	Baseline	6-month follow-up	*P*-value	Baseline	6-month follow-up	*P*-value	Baseline	6-month follow-up	*P*-value
Left heart									
LAVI, mL/m^2^	52.8 (39.7, 72.0)	44.9 (30.2, 52.4)	0.009	51.5 (38.8, 79.4)	47.2 (30.2, 53.1)	0.005	53.3 (42.0, 55.4)	41.0 (29.6, 48.3)	0.161
E/A ratio	1.6 ± 1.1	1.5 ± 0.9	0.523	1.5 ± 1.0	1.2 ± 0.7	0.206	1.6 ± 1.0	1.3 ± 0.7	0.345
E/e′ ratio	12.7 ± 5.2	13.5 ± 5.2	0.641	14.2 ± 6.1	12.7 ± 5.1	0.197	10.5 ± 3.3	12.9 ± 3.7	0.263
Mitral regurgitation, % (n)			0.051			0.627			0.058
None/Trace	14.3% (4/28)	25.0% (7/28)		20.0% (4/20)	20.0% (4/20)		0.0% (0/8)	37.5% (3/8)	
Mild	17.9% (5/28)	17.9% (5/28)		15.0% (3/20)	15.0% (3/20)		25.0% (2/8)	25.0% (2/8)	
Moderate	32.1% (9/28)	39.3% (11/28)		25.0% (5/20)	45.0% (9/20)		50.0% (4/8)	25.0% (2/8)	
Severe	35.7% (10/28)	17.9% (5/28)		40.0% (8/20)	20.0% (4/20)		25.0% (2/8)	12.5% (1/8)	
LVEDVI, mL/m^2^	103.7 ± 35.1	106.0 ± 43.7	0.811	121.5 ± 45.2	122.0 ± 50.0	0.938	77.4 ± 22.7	71.1 ± 15.3	0.161
LVESVI, mL/m^2^	69.1 ± 30.0	69.5 ± 41.2	0.362	80.3 ± 28.5	77.6 ± 32.9	0.624	41.1 ± 15.2	38.0 ± 15.2	0.161
LVEF, %	34.5 (29.0, 39.7)	35.0 (28.5, 47.9)	0.733	30.2 (29.0, 35.0)	30.8 (26.6, 38.0)	0.072	47.0 (39.9, 51.2)	45.5 (37.3, 58.3)	0.484
LVGLS, %	−10.6 (−11.6, −10.3)	−14.3 (−14.5, −14.2)	<0.001	−10.5 (−10.8, −10.2)	−14.3 (−14.5, −14.2)	<0.001	−12.1 (−12.5, −10.5)	−14.2 (−14.4, −12.4)	0.161
Right heart									
RA dimension, mm	39.2 ± 7.6	43.9 ± 8.0	0. 002	40.1 ± 8.1	43.6 ± 8.1	0. 036	37.0 ± 6.0	44.6 ± 8.1	0.017
RV dimension, mm	39.2 ± 8.5	39.7 ± 7.5	0.346	38.7 ± 8.8	39.5 ± 7.0	0.657	40.4 ± 8.2	40.3 ± 9.1	0.865
Tricuspid regurgitation, % (n)			0.869			0.331			0.655
None/Trace	28.6% (8/28)	17.9% (5/28)		30.0% (6/20)	20.0% (4/20)		25.0% (2/8)	12.5% (1/8)	
Mild	25.7% (10/28)	46.4% (13/28)		40.0% (8/20)	45.0% (9/20)		25.0% (2/8)	50.0% (4/8)	
Moderate	10.7% (3/28)	17.9% (5/28)		5.0% (1/20)	15.0% (3/20)		25.0% (2/8)	25.0% (2/8)	
Severe	25.0% (7/28)	17.9% (5/28)		25.0% (5/20)	20.0% (4/20)		25.0% (2/8)	12.5% (1/8)	
S′ velocity, cm/s	6.0 (4.9, 6.8)	6.2 (5.0, 7.4)	0585	6.1 (4.9, 7.0)	5.8 (5.0, 7.4)	0.561	5.8 (4.8, 6.7)	6.4 (5.1, 8.0)	0.674
TAPSE, mm	17.0 ± 3.1	17.0 ± 2.9	0.652	16.8 ± 3.0	17.0 ± 3.0	0.059	18.0 ± 3.2	17.0 ± 3.4	0.222
RVFAC, %	40.5 ± 11.6	39.0 ± 10.3	0.425	37.7 ± 12.4	38.3 ± 10.4	0.102	46.5 ± 10.3	43.6 ± 6.7	0.263
RVFWLS, %	−15.6 (−17.0, −15.4)	−18.8 (−19.8, −17.9)	<0.001	−15.5 (−15.6, −15.2)	−18.5 (−19.5, −18.0)	<0.001	−17.4 (−17.5, −17.1)	−21.7 (−23.6, −16.3)	0.063

Values are presented in the form mean ± SD, % (n), or median (interquartile range). LAVI, left atrial volume index; E, mitral inflow peak early diastolic velocity; A, peak late diastolic velocity; e′, septal and lateral mitral annular early diastolic velocities; LVEDVI, left ventricular end-diastolic volume index; LVESVI, left ventricular end-systolic volume index; LVEF, left ventricular ejection fraction; LVGLS, left ventricular global longitudinal strain; RA, right atrial; RV, right ventricular; S′, tricuspid lateral annular systolic velocity; RVFAC, right ventricular fractional area change; TAPSE, tricuspid annulus plane systolic excursion; RVFWLS, right ventricular free wall longitudinal strain.

**Figure 2:**
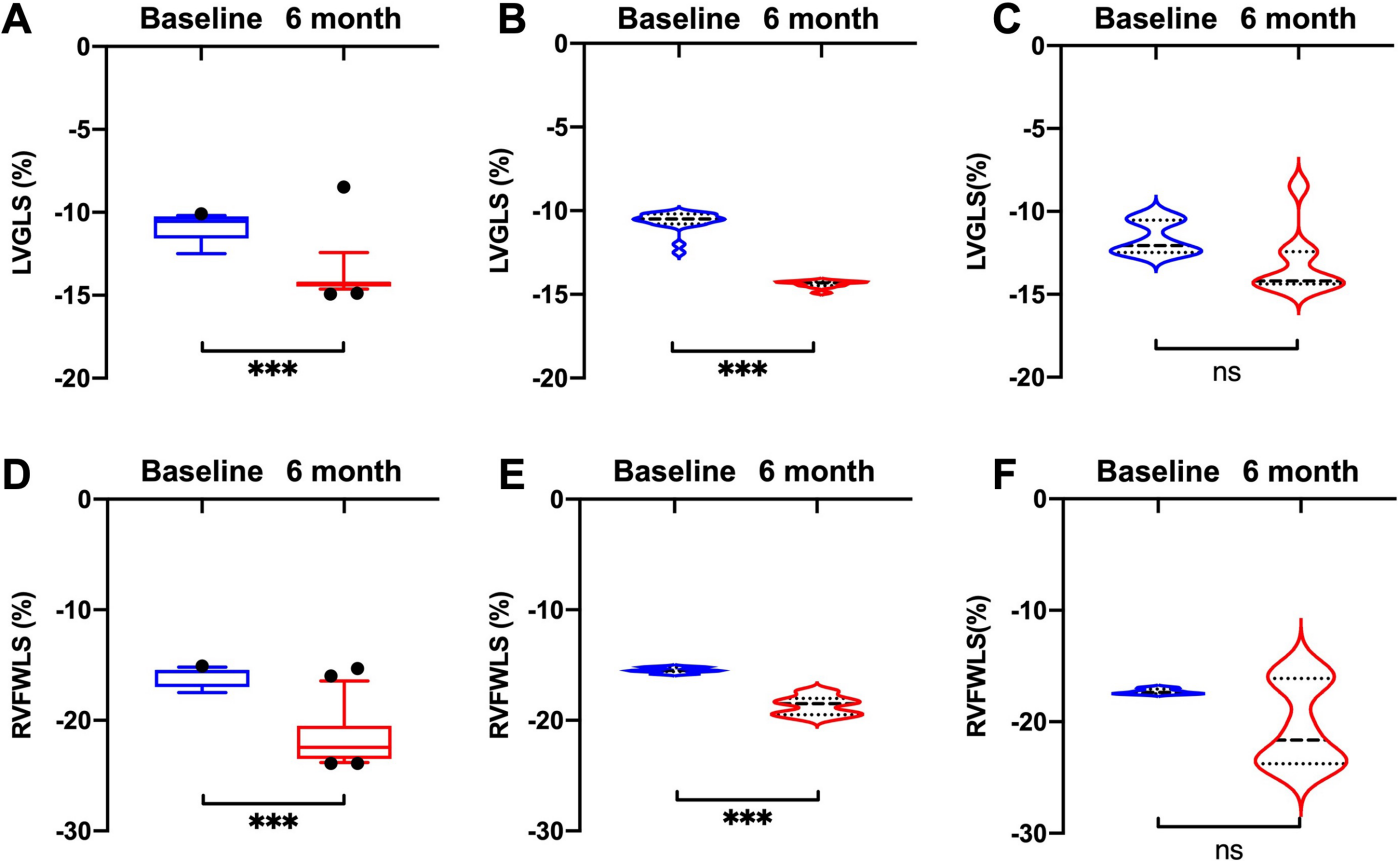
Comparisons of LVGLS and RVFWLS values at baseline and 6 months after D-shant device implantation. (**A**) LVGLS and (**D**) RVFWLS in all patients; (**B**) LVGLS and (**E**) RVFWLS in patients with HFrEF; and (**C**) LVGLS and (**F**) RVFWLS in patients with HFpEF. HFpEF, heart failure with preserved ejection fraction; HFrEF, heart failure with reduced ejection fraction; LVGLS, left ventricular global longitudinal strain; RVFWLS, right ventricular free wall longitudinal strain.

Compared with baseline measurements, significant improvements in LVGLS and RVFWLS were observed in the HFrEF group (LVGLS: −10.5 [−10.8, −10.2] vs. −14.3 [−14.5, −14.2], *P *< 0.001; RVFWLS: −15.5 [−15.6, −15.2] vs. −18.5 [−19.5, −18.0], *P *< 0.001) (Figure 2), but not in the HFpEF group.

### Predictors of functional improvement after implantation of D-shant device

**Figure 3:**
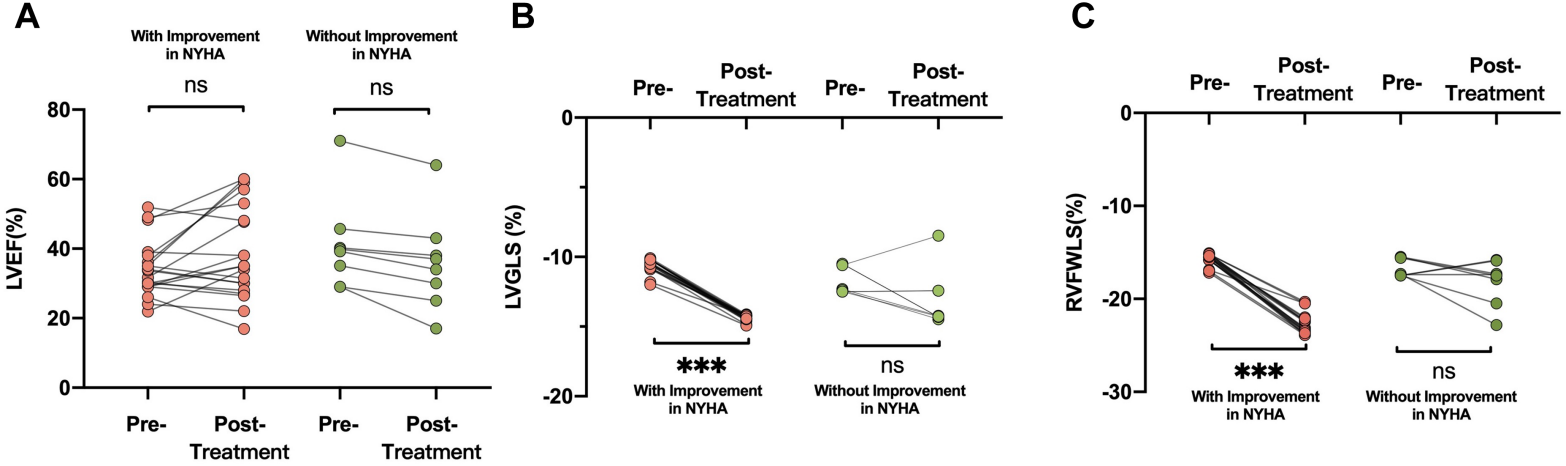
Comparisons of (**A**) LVEF, (**B**) LVGLS, and (**C**) RVFWLS in patients who did and did not undergo improvement in NYHA functional class after D-shant device implantation. LVEF, left ventricular ejection fraction; NYHA, New York Heart Association; LVGLS, left ventricular global longitudinal strain; RVFWLS, right ventricular free wall longitudinal strain.

An improvement in NYHA functional class at 6 months after implantation of the D-Shant device was noted in 17 of 20 patients with HFrEF, and in 3 of 8 patients with HFpEF. The baseline echocardiographic characteristics of patients with and without functional improvements in NYHA functional class are shown in Table 3. Compared with patients who did not show improvement in NYHA functional class, those who did show improvement exhibited lower absolute values of LVGLS and RVFWLS at baseline (Figure 3). However, LAVI, LVEDVI, LVESVI, LVEF, E/A, E/e′, RA and RV dimensions, tricuspid S′, TAPSE, RVFAC, and degree of mitral and tricuspid regurgitation did not differ between the two groups.

**Table 3:** Baseline echocardiographic characteristics in patients who did and did not undergo improvement in NYHA functional class after D-shant device implantation.

Parameter	Patients with improvement in NYHA functional class (*n* = 20)	Patients without improvement in NYHA functional class (*n* = 8)	*P*-value
Left heart			
LAVI, mL/m^2^	53.1 (41.0, 81.0)	46.6 (33.1, 55.3)	0.237
LVEDVI, mL/m^2^	106.9 ± 32.0	95.8 ± 43.3	0.354
LVESVI, mL/m^2^	72.9 ± 27.9	59.6 ± 34.8	0.218
LVEF, %	32.7 (29.0, 37.5)	39.5 (30.5, 44.3)	0.123
E/A ratio	1.7 ± 1.2	1.3 ± 0.9	0.262
E/e′ ratio	13.7 ± 5.2	10.2 ± 4.5	0.123
Mitral regurgitation, % (*n*)			0.202
None/Trace	20.0% (4/20)	0.0% (0/8)	
Mild	10.0% (2/20)	37.5% (3/8)	
Moderate	35.0% (7/20)	25.0% (2/8)	
Severe	35.0% (7/20)	37.5% (3/8)	
LVGLS, %	−10.5 (−10.8, −10.2)	−12.4 (−12.5, −10.5)	0.006
Right heart			
RA dimension, mm	40.2 ± 8.1	36.8 ± 5.8	0.237
RV dimension, mm	40.0 ± 9.5	37.1 ± 5.2	0.566
S′ velocity, cm/s	6.0 (4.8, 7.0)	6.1 (5.2, 6.7)	0.862
TAPSE, mm	17.0 ± 3.2	17.1 ± 3.0	0.980
RVFAC, %	37.9 ± 11.6	46.9 ± 9.5	0.070
Tricuspid regurgitation, % (*n*)			0.743
None/Trace	20.0% (4/20)	50.0% (4/8)	
Mild	40.0% (8/20)	25.0% (2/8)	
Moderate	10.0% (2/20)	12.5% (1/8)	
Severe	30.0% (6/20)	12.5% (1/8)	
RVFWLS, %	−15.5 (−15.8, −15.2)	−17.4 (−17.5, −15.5)	0.010

Values are presented in the form mean ± SD, % (*n*), or median (interquartile range). LAVI, left atrial volume index; E, mitral inflow peak early diastolic velocity; A, peak late diastolic velocity; e′, septal and lateral mitral annular early diastolic velocities; LVEDVI, left ventricular end-diastolic volume index; LVESVI, left ventricular end-systolic volume index; LVEF, left ventricular ejection fraction; LVGLS, left ventricular global longitudinal strain; RA, right atrial; RV, right ventricular; S′, tricuspid lateral annular systolic velocity; RVFAC, right ventricular fractional area change; TAPSE, tricuspid annulus plane systolic excursion; RVFWLS, right ventricular free wall longitudinal strain.

To investigate the predictors of improvement in NYHA functional class, we performed univariate and multivariate analyses (Table 4). Univariate logistic regression analysis showed that 6 MWT score [odds ratio (OR): 1.025; 95% CI: 1.003–1.047; *P* = 0.028], LVGLS (OR: 5.788; 95% CI: 1.673–20.029; *P* = 0.006), RVFAC (OR: 4.300; 95% CI: 1.427–12.955; *P* = 0.010), and RVFWLS (OR: 2.640; 95% CI: 1.464–4.762; *P* = 0.001) were predictors of improvement in NYHA functional class at 6-month follow-up after device implantation among all patients with HF. In contrast, age, gender, NT-proBNP, PCWP, LAVI, LVEF, RA and RV dimensions, and TAPSE were not predictive of NYHA functional class improvement. In multivariate models, 6 MWT score and KCCQ score continued to be of prognostic value. LVGLS (OR: 5.930; 95% CI: 1.463–24.038; *P* = 0.013) and RVFWLS (OR: 4.852; 95% CI: 1.372–17.159; *P* = 0.014) were associated with improvement in NYHA functional class.

**Table 4:** Univariate and multivariate analyses of factors associated with improvement in NYHA functional class after D-shant device implantation.

			Model 1		Model 2	
	Univariate		6 WMT + KCCQ + LVGLS		6 WMT + KCCQ + RVFWLS + RVFAC	
	OR (95% CI)	*P*-value	OR (95% CI)	*P*-value	OR (95% CI)	*P*–value
Age	0.920 (0.833–1.016)	0.101				
Gender	0.600 (0.112–3.214)	0.551				
NT-proBNP	1.000 (1.000–1.000)	0.331				
6 MWT	1.025 (1.003–1.047)	0.028	1.023 (1.001–1.046)	0.041		0.254
KCCQ	1.150 (0.999–1.323)	0.052		0.102	1.156 (1.005–1.330)	0.043
PCWP	1.561 (0.913–2.669)	0.104				
LAVI	1.040 (0.990–1.093)	0.117				
LVEF	0.932 (0.850–1.021)	0.128				
LVGLS	5.788 (1.673–20.029)	0.006	5.930 (1.463–24.038)	0.013		
RA dimension	2.073 (0.548–7.846)	0.283				
RV dimension	1.579 (0.535–4.661)	0.408				
TAPSE	0.985 (0.752–1.292)	0.915				
RVFAC	4.300 (1.427–12.955)	0.010				0.074
RVFWLS	2.640 (1.464–4.762)	0.001			4.852 (1.372–17.159)	0.014

Odds ratios are presented with 95% CIs.

LAVI, left atrial volume index; LVEF, left ventricular ejection fraction; LVGLS, left ventricular global longitudinal strain; RA, right atrial; RV, right ventricular; RVFAC, right ventricular fractional area change; TAPSE, tricuspid annulus plane systolic excursion; RVFWLS, right ventricular free wall longitudinal strain; PCWP, pulmonary capillary wedge pressure; NT-proBNP, N-terminal pro-B type natriuretic peptide; 6 MWT, 6-min walk test; KCCQ, Kansas City Cardiomyopathy Questionnaire.

**Figure 4:**
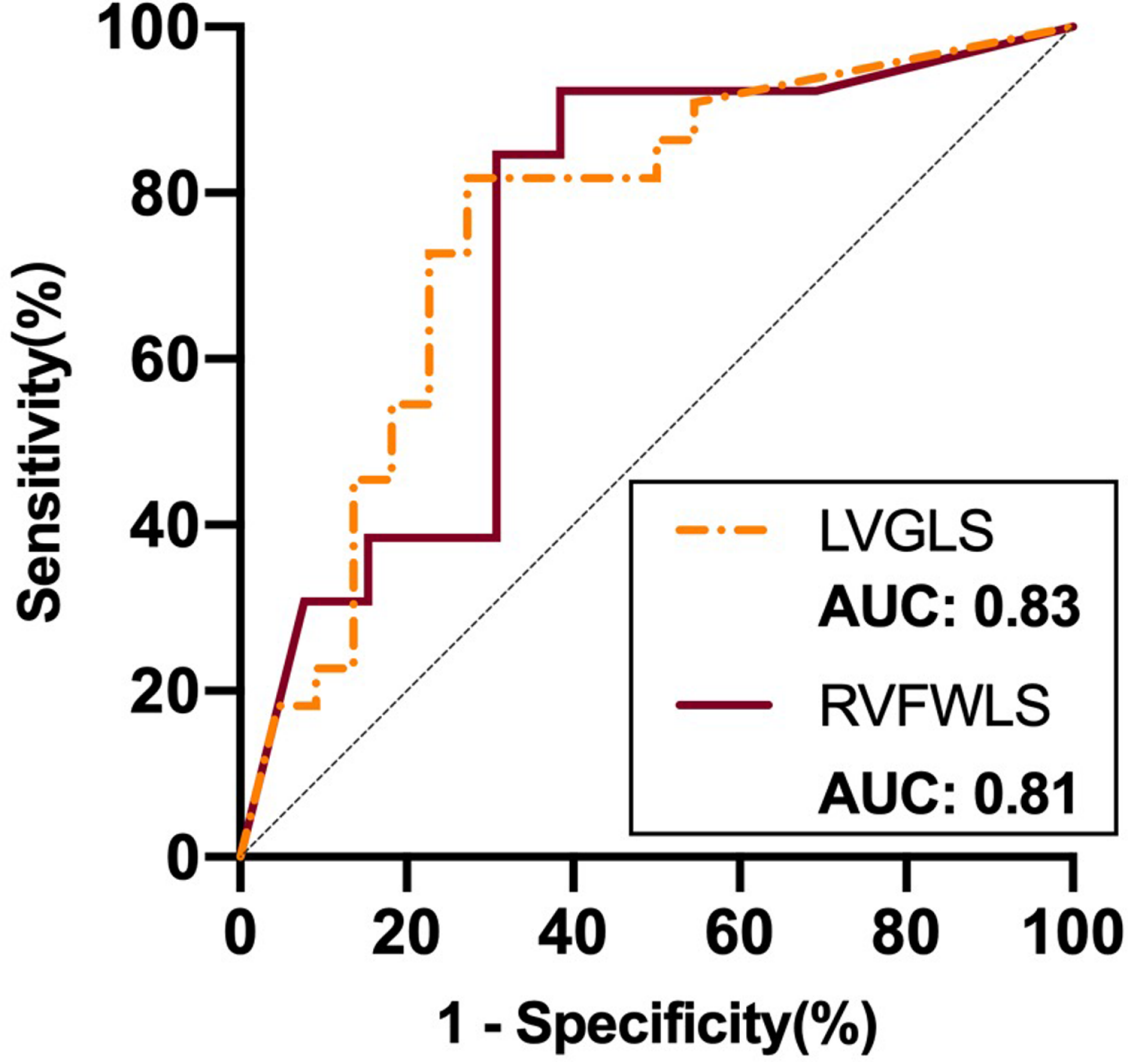
ROC curve analysis of STE parameters as predictors of improvement in NYHA functional class after D-shant device implantation. ROC, receiver-operating characteristic; NYHA, New York Heart Association; LVGLS, left ventricular global longitudinal strain; RVFWLS, right ventricular free wall longitudinal strain.

ROC curve analysis of these variables for use in prediction of improvement in NYHA functional class showed that LVGLS (area under the curve: 0.83) and RVFWLS (area under the curve: 0.81) were associated with NYHA functional class improvement at 6 months after implantation (Figure 4). The optimal cutoff value for LVGLS for prediction of this outcome was −12.15%, with sensitivity of 100% and specificity of 63%; the best cutoff value for RVFWLS was −17.25% (sensitivity: 100%; specificity: 63%).

### Reproducibility

Intra-observer and inter-observer reproducibility are shown in Table 5. The inter-observer and intra-observer ICCs for LVGLS and RVFWLS were all greater than 0.90, indicating that both parameters exhibited excellent reproducibility. Inter-observer and intra-observer agreement for LVGLS and RVFWLS were high, as reflected by the small bias values and narrow limits of agreement.

**Table 5:** The reproducibility of LVGLS and RVFWLS measurements.

	ICC (95% CI)	Bias	Limits of agreement
Intra-observer			
LVGLS, %	0.89 (0.82–0.93)	−0.07	−4.07–3.92
RVFWLS, %	0.99 (9.6–0.10)	−0.29	−1.16–0.58
Inter-observer			
LVGLS, %	0.92 (0.87–0.95)	−0.14	−3.43–3.16
RVFWLS, %	0.99 (9.7–1.00)	−0.27	−1.12–0.66

CI, confidence interval; LVGLS, left ventricular global longitudinal strain; ICC, intraclass correlation coefficient; RVFWLS, right ventricular free wall longitudinal strain.

## Discussion

This study indicated that preoperative LVGLS and RVFWLS are associated with improvement in the NYHA functional class of patients 6 months after implantation of an interatrial shunt device. Specifically, patients who experienced improvement in their NYHA functional class had lower absolute values of LVGLS and RVFWLS. The significance of these findings is that they suggest these novel parameters could be useful in selecting appropriate patients for atrial shunt device implantation. This could have a significant impact on clinical decision-making, as doctors may now be able to use LVGLS and RVFWLS values to identify patients who are more likely to benefit from this intervention. Additionally, the ability to predict which patients may experience functional improvement following the procedure could help doctors to counsel patients more effectively on the potential benefits and risks of atrial shunt device implantation. Overall, this study contributes new knowledge to the field and has the potential to positively impact patient outcomes.

A significant reduction in LAVI was observed in the HFrEF group at 6-month follow-up, suggesting an adaptive change in LA volume in response to the interatrial shunt device. Previous studies have indicated that LA volume is a predictor of cardiac adverse events in HF patients (22, 23). In addition, an increase in RA size was noted in our study, which could represent an effect of the shunting itself or reflect an increase in circulating volume. These findings were in line with those obtained for the Corvia IASD, which has been shown to be associated with significant enlargement in RA volume at 6 months (9).

Consistent with previous work (10, 24), there was evidence of alleviation of symptoms and improved quality of life at 6-month follow-up. Improvement in NYHA functional class was observed in 85.0% of patients (17/20) with HFrEF, which is in accordance with previous reports (17, 24). In contrast, 62.5% (5/8) of patients with HFpEF were unchanged in terms of NYHA functional class at 6-month follow-up. Similarly to this finding, the REDUCE LAP-HF I trial indicated no significant improvement in NYHA functional class in patients with HFpEF after interatrial shunt device implantation (25). Plausible explanations might be greater myocardial damage and pulmonary disease (9), and further research is warranted to systematically test these results.

2D-STE allows for the semiautomated quantification of myocardial mechanics and helps to refine the decision-making process in patients with HF. However, evidence on the clinical implications of 2D-STE measurements in patients with interatrial shunt device implantation is scarce. Our study has revealed the differentiation in changes in ventricular function response to interatrial shunt device implantation between patients with HFrEF and those with HFpEF. Improvements in LVGLS and RVFWLS were identified in HFrEF patients, but not in the HFpEF group. In patients with HFrEF, LV unloading caused by device implantation could account for the higher value of LVGLS at follow-up (26). Similarly, the greater RVFWLS after implantation of the device can be attributed to compensatory “overwork” of the RV myocardium, owing to the large preload reserve of the RV and pulmonary vascular reserve (27). Patients with HFpEF frequently have biventricular components to their cardiomyopathy and virtually always have abnormalities in pulmonary arterial capacitance and some degree of pulmonary vascular remodeling (12, 28, 29), rendering them more sensitive to even minor degrees of RV volume overload. Our findings are novel in that we have identified LVGLS and RVFWLS as predictors of improvement in NYHA functional class after interatrial shunt device implantation. Elshafey et al. have recently documented the significant correlation of LVGLS with NYHA functional class among patients with HFrEF (30). Another study has found that LV segmental longitudinal strain could be a predictor of recovery of cardiac function in patients with reduced LVEF (15). In our cohort, biventricular systolic myocardial deformation may have been associated with recovery of cardiac function after D-Shant device implantation. Myocardial systolic strain has been linked to serum levels of aminoterminal propeptide of procollagen I/III and tissue inhibitor of matrix metalloproteinase-1, both of which are indicators of cardiac fibrosis (31). Biventricular longitudinal strain may prove to be a noninvasive surrogate measure for prediction of NYHA functional class improvement in patients who undergo interatrial shunt device implantation. Although this study suggests an association between biventricular longitudinal strain and recovery of cardiac function, the results should be further tested in a large cohort of patients with HF.

### Limitations

Because of the strict exclusion criteria and the fact that this was a small single-center study, the sample size was small. We were unable to include several clinical variables (e.g., cardiovascular diseases, RAP, and PCWP-RAP) in the model due to the small number of cases, which raises the possibility of model overfit. As a result, we were unable to modify our model fully. The current study should be viewed as a proof of concept, and larger multicenter investigations are needed. Additionally, invasive right heart catheterization was not performed in our cohort of patients at 6-month follow-up. Although our results indicated biventricular longitudinal strain on echocardiography to be a valuable predictor of NYHA functional class improvement after interatrial shunt device implantation among patients with HFrEF and HFpEF, the diagnostic utility of strain cutoffs as noninvasive markers was not provided owing to the small sample size. Future research is warranted to test this issue, as well as the relative clinical utility of biventricular longitudinal strain in larger-scale epidemiological cohorts.

## Conclusions

The findings of this study suggest that biventricular longitudinal strain can be used as a non-invasive surrogate measure for prediction of recovery of cardiac function after implantation of a D-Shant device. This information may have a significant impact on clinical decision-making by enabling physicians to identify patients who are likely to benefit from the device and to make more informed decisions regarding atrial shunt device implantation.

Overall, this study has the potential to improve patient outcomes and reduce morbidity and mortality in heart failure patients, making it a valuable contribution to the field of cardiology.

## Data Availability

The original contributions presented in the study are included in the article/**Supplementary Material**, further inquiries can be directed to the corresponding author/s.
