# Supplementary material for: Biventricular longitudinal strain as a predictor of functional improvement after D-shant device implantation in patients with heart failure
Source: Front Cardiovasc Med. 2023 Apr 17;10:1121689. doi: 10.3389/fcvm.2023.1121689 (PMC10149702; doi:10.3389/fcvm.2023.1121689)

Supplemental Information

Figure 1 Changes in symptomatic and functional state at baseline and 6 months after D-Shant device implantation. (A) NYHA functional class. (B) 6 MWT. (C) KCCQ score. NYHA, New York Heart Association; 6 MWT, 6-min walk test; KCCQ, Kansas City Cardiomyopathy Questionnaire.

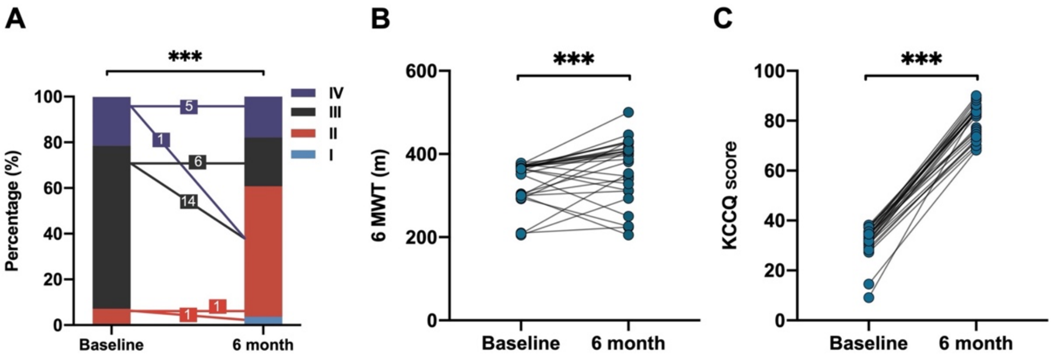

Supplement: Supplementary file 1 [file Image1.pdf]
