# Supplementary material for: Biventricular longitudinal strain as a predictor of functional improvement after D-shant device implantation in patients with heart failure
Source: Front Cardiovasc Med. 2023 Apr 17;10:1121689. doi: 10.3389/fcvm.2023.1121689 (PMC10149702; doi:10.3389/fcvm.2023.1121689)
Supplement: Supplementary file 2 [file Table1.pdf]

Supplemental Information

Table 1. Specifications/Model of D-Shant device

Number	Gender	Age(y)	BMI	Specification/Model
1	M	56	24.82	WKASD20-6
2	F	47	22.83	WKASD24-8
3	F	69	22.1	WKASD24-8
4	F	55	25.33	WKASD24-8.
5	M	73	23.5	WKASD24-6
6	F	57	26.26	WKASD24-8
7	M	40	25.95	WKASD24-8
8	M	61	18.14	WKASD20-6
9	M	36	23.39	WKASD24-8
10	M	72	26.33	WKASD20-6
11	M	69	23.15	WKASD24-8.
12	F	56	20.58	WKASD24-8.
13	M	29	20.42	WKASD24-8
14	F	76	17.97	WKASD24-8
15	F	67	26.56	WKASD28-10
16	M	53	32.15	WKASD24-8
17	M	59	20.28	WKASD24-6
18	F	73	18.03	WKASD24-8
19	F	69	18.99	WKASD24-8
20	F	58	22.6	WKASD24-8
21	M	53	22.68	WKASD20-6
22	M	58	26.03	WKASD20-6
23	M	59	26.85	WKASD24-8
24	F	71	21.64	WKASD24-8
25	F	51	17.48	WKASD20-6
26	M	69	22.77	WKASD20-6
27	M	65	24.22	WKASD24-8
28	M	50	20.08	WKASD24-8
29	M	64	37.76	WKASD24-8
30	F	71	23.5	WKASD12-8
31	M	71	21.48	WKASD20-6
32	F	55	18.73	WKASD16-4
33	F	42	26.04	WKASD20-6
34	F	53	28.76	WKASD16-4

M, male; F, female; BMI, body mass index; WKASD, WeiKe medical Atrial Shunt Device; 20-6, 20 mm near surface diameter and 6 mm shunt aperture
